# Biallelic variants in *LARS1* induce steatosis in developing zebrafish liver via enhanced autophagy

**DOI:** 10.1186/s13023-024-03226-6

**Published:** 2024-05-28

**Authors:** Masanori Inoue, Wulan Apridita Sebastian, Shota Sonoda, Hiroaki Miyahara, Nobuyuki Shimizu, Hiroshi Shiraishi, Miwako Maeda, Kumiko Yanagi, Tadashi Kaname, Reiko Hanada, Toshikatsu Hanada, Kenji Ihara

**Affiliations:** 1 Department of Pediatrics, Oita University Faculty of Medicine, Oita, Japan; 2 Department of Neuropathology, Institute for Medical Science of Aging, Aichi Medical University, Aichi, Japan; 3 Department of Cell Biology, Oita University Faculty of Medicine, Oita, Japan; 4 Department of Genome Medicine, National Center for Child Health and Development, Tokyo, Japan; 5 Department of Neurophysiology, Oita University Faculty of Medicine, Oita, Japan

**Keywords:** Autophagy, DGAT1, LARS1, Fatty liver, ILFS1

## Abstract

**Background:**

Biallelic pathogenic variants of *LARS1* cause infantile liver failure syndrome type 1 (ILFS1), which is characterized by acute hepatic failure with steatosis in infants. LARS functions as a protein associated with mTORC1 and plays a crucial role in amino acid-triggered mTORC1 activation and regulation of autophagy. A previous study demonstrated that *larsb*-knockout zebrafish exhibit conditions resembling ILFS. However, a comprehensive analysis of *larsb*-knockout zebrafish has not yet been performed because of early mortality.

**Methods:**

We generated a long-term viable zebrafish model carrying a *LARS1* variant identified in an ILFS1 patient (*larsb-I451F* zebrafish) and analyzed the pathogenesis of the affected liver of ILFS1.

**Results:**

Hepatic dysfunction is most prominent in ILFS1 patients during infancy; correspondingly, the *larsb-I451F* zebrafish manifested hepatic anomalies during developmental stages. The *larsb-I451F* zebrafish demonstrates augmented lipid accumulation within the liver during autophagy activation. Inhibition of DGAT1, which converts fatty acids to triacylglycerols, improved lipid droplets in the liver of *larsb-I451F* zebrafish. Notably, treatment with an autophagy inhibitor ameliorated hepatic lipid accumulation in this model.

**Conclusions:**

Our findings suggested that enhanced autophagy caused by biallelic *LARS1* variants contributes to ILFS1-associated hepatic dysfunction. Furthermore, the *larsb-I451F* zebrafish model, which has a prolonged survival rate compared with the *larsb*-knockout model, highlights its potential utility as a tool for investigating the pathophysiology of ILFS1-associated liver dysfunction.

**Supplementary Information:**

The online version contains supplementary material available at 10.1186/s13023-024-03226-6.

## Introduction

Infantile liver failure syndrome type 1 (ILFS1; OMIM 615438) is a rare autosomal recessive disorder caused by pathogenic variants of the *LARS1* gene (OMIM 151350) located on chromosome 5q32, which encodes leucyl-tRNA synthetase (LARS) and catalyzes the ligation of leucine to leucine tRNA [1]. This syndrome was initially reported in 2012 within the Irish Traveller community with a predominant hepatic phenotype [1, 2].

ILFS1 is clinically characterized by recurrent liver dysfunction, intrauterine growth retardation, failure to thrive, neurodevelopmental delay, encephalopathy, microcytic anemia, hypoalbuminemia, coagulation disorders, and muscular hypotonia [3]. Major symptoms appear from the fetal period to infancy, and some of them, such as liver dysfunction, neurodevelopmental delay, or muscular hypotonia, have been reported to recover or disappear spontaneously in early childhood [3].

Acute liver failure (ALF) in ILFS1 is a life-threatening event [3, 4]. Liver transaminase elevation is typically observed from the neonatal to pediatric period in most patients, often progressing to fulminant liver failure triggered by febrile illnesses within two years after birth [3]. A histological evaluation of liver biopsy samples during ALF episodes revealed remarkable fatty degeneration [3]. Nutritional management with high-protein diets and leucine supplementation or prophylactic antipyretic therapy and vaccination was recommended in some reports on a case-by-case basis by experts [3, 5]. Liver transplantation may be the final option for ALF; however, the transplanted allograft liver never resolves the damage to other organs, and ALF is expected to recover spontaneously. Evidence-based management and treatment of ILFS1 in the liver during infancy thus remain to be established [3].

The LARS protein encoded by the *LARS1* gene is ubiquitously expressed and serves as an essential enzyme in protein synthesis, catalyzing the ligation of amino acids to their cognate transfer RNAs and marking the initial step of aminoacyl-tRNA synthesis [6, 7]. LARS is a class I enzyme, characterized by the Rossman fold, a large-insertion CP1 domain, a tRNA-binding anticodon domain, and a C-terminal extension domain [8]. In higher eukaryotes, LARS is a part of the multi-tRNA synthetase complex, consisting of nine tRNA synthetases and three nonenzymatic components [9–11]. LARS catalyzes the binding of leucine to leucine tRNA using adenosine triphosphate [6, 7]. Furthermore, LARS plays a unique non-canonical role as a mammalian target of rapamycin complex 1 (mTORC1)-associated protein required for amino acid-induced mTORC1 activation, which acts as an intracellular leucine sensor for mTORC1 signaling [6, 7, 12, 13]. mTORC1 regulates protein synthesis, autophagy, and cell growth [14–16]. Thus, LARSs play broad roles in cellular homeostasis, including translational control, transcriptional regulation, tumorigenesis, and senescence [6, 7, 12, 13, 17, 18].

Our previous research using *larsb*-knockout zebrafish demonstrated that mutant zebrafish exhibit a phenotype similar to that of ILFS1 [19]. Excessive autophagy activation was observed in *larsb*-knockout zebrafish, and the suppression of autophagy by bafilomycin treatment significantly recovered the liver size and improved the survival curve [19]. However, early lethality, probably due to severe liver damage, nervous system disorders, and anemia in *larsb*-knockout larvae, did not allow us to analyze the exact molecular mechanism by which LARS pathogenic variants affect the development and function of the liver in ILFS1 patients.

To further evaluate the role of LARS and the effects of its defect in the pathogenesis of the liver, we generated *larsb*-knockin zebrafish with a biallelic missense variant of the *LARS1* gene identified in an ILFS1 patient in our hospital. We investigated the molecular function of Lars in the context of ILFS1 pathogenesis.

## Materials and methods

### WES (whole-exome sequencing) and filtering analyses

Genomic DNA was extracted from the peripheral blood of the proband, his sister, and the parents using a QIA amp DNA blood mini kit (QIAGEN, Venlo, Netherlands) and sequenced by WES. WES and variant filtering analyses were performed as previously described with slight modifications [20]. In brief, after sharing the DNA with a Covaris Focused-ultrasonicator S220 (Woburn, MA, USA), the sequence library was prepared using a Human All Exon V6 Kit (Agilent Technologies, Santa Clara, CA, USA) and sequenced using a 2500 Illumina with 125-bp paired-end reads (Illumina, San Diego, CA, USA). Sequence reads were aligned to GRCh38 and annotated using CompStor NOVOS and CompStor Insight, which carry a proprietary Novos caller and annotation engine (OmniTier, San Jose, CA, USA). The filtering procedure for the annotated variants is as follows: first, variants with allele frequencies > 0.01 were removed from the public databases gnomAD and 14 KJPN (jMORP) as well as our in-house exome variant data consisting of more than 7,000 WES data. Next, the variants were narrowed down based on assumed modes of inheritance, such as autosomal dominant, autosomal recessive, X-linked, and compound heterozygous. Finally, two variants of *LARS1* and one variant of *LAMA4* were co-segregated, with the latter variant not exhibiting concordant clinical symptoms (S1 Table). No pathogenic copy number variations were detected in the WES data. The two *LARS1* variants were confirmed by Sanger sequencing (ABI3130) using the primers 5′- GGGTCTCATAACAATGAATACTTC -3′ and 5′- GGGAAAAGGTAGGCTACAAGG -3’ for NM_020117:c.601 T > G and 5′- GGCAGTGTCGTAATGACATATAC-3′ and 5′-CCATAGAGATTCCTAGAGGG-3′ for c.1351A > T.

### Zebrafish maintenance

The *larsb* mutant and Tg[*fabp10*:mcherry] zebrafish AB genetic background were raised and maintained following standard procedures [21, 22]. They were maintained at 28–29 °C under a 14-h:10-h light:dark cycle. Embryos were collected and housed at 28.5 °C.

All animal experimental procedures were performed in accordance with the institutional and national guidelines and regulations. The study was conducted in compliance with the ARRIVE guidelines.

### Generation of the larsb I451F zebrafish line

The *larsb I451F* zebrafish line was generated via CRISPR/ Cas9 gene editing [23, 24]. The site of the *larsb* sgRNA target was 5′-CCAAAGCCAGAATGACAGAGAGA-3′ in the editing domain of the LARS protein. Single-stranded oligodeoxynucleotides (ssODNs) were designed with the following sequences (phosphorothioate modifications in the first and last nucleotides) and ordered as ultramers by Integrated DNA Technologies (Coralville, IA, USA) to generate single-nucleotide polymorphisms: A*G*TGGCTTATTGGTTTGTTCTACCAGGTTCCCATCATTGAAATTCCAGGGTATGGGAATCTGTCAGCTCCACTGGTGTGCGATGAACTGAAGTTTCAAAGCCAGAATGACAGAGAGAAACTGGCCGAGG*C*T. The microinjection solution (1 nl; Cas9 protein [300 pg], gRNA [30 pg], ssODNs [41 pg], and 0.1% phenol red) was microinjected into single-cell-stage wild-type (WT) embryos. The microinjected zebrafish were raised to adulthood and screened for germline transmission of *larsb* mutations through natural breeding. We crossed adult microinjected zebrafish with WT zebrafish and collected 20 embryos into a single polymerase chain reaction (PCR) tube, which was then heated at 95 °C for 15 min in 180 μL of 50 mM NaOH. Subsequently, 20 μL of 1 M Tris–HCl was added to each sample. The DNA extraction sample was used as a template, and PCR was performed using the *larsb I451F* forward primer 5'-TGTGCGATGAACTGAAGTTT-3' and *larsb* reverse primer 5'- CACATCTCCTTTCATGCGTTT-3,’ with GoTaq DNA polymerase (Promega, WI, USA). These primers were designed to specifically recognize knock-in sequences. The PCR program was as follows: 95 °C for 2 min, followed by 35 cycles at 95 °C for 30 s, 63 °C for 30 s, and 72 °C for 12 s. Targeted mutations were verified by Sanger sequencing of PCR-positive zebrafish embryo DNA obtained from genotyped PCR. The primers used for Sanger sequencing were the *larsb* forward primer 5'-TCATGCCAAGTCAAGTCCTG-3' and *larsb* reverse primer. The F0 founder, with germline transmission, was selected to establish a knock-in zebrafish line. The F1 generations were raised to adulthood, had their fins clipped, and were sequenced. Consequently, a homozygous *larsb I451F* zebrafish line (*larsb I451F* zebrafish line) was identified.

### Generation of transgenic zebrafish

Tg[*fabp10*:mCherry] fish expressing mCherry exclusively in hepatocytes were generated using a MultiSite Gateway kit (Thermo Fisher Scientific, Waltham, MA, USA) to produce vectors with Tol2 transposon sites [25]. A 2.8-kb promoter of the zebrafish *fabp10* gene [21] was amplified from genomic DNA in WT zebrafish by PCR (KOD-plus-Neo; Toyobo, Osaka, Japan). The PCR primers used were the *fabp10* forward primer 5'-AAAAAGCTTGCAGTAAATTGATTCAAACT-3' and *fabp10* reverse primer 5'-AAAGGATCCGCTTTCTGGAGAAGCTCAAC-3'. The PCR program was as follows: 94 °C for 2 min, followed by 30 cycles at 98 °C for 10 s, 60 °C for 30 s, and 68 °C for 90 s. The PCR mixture was subjected to agarose gel electrophoresis, and the desired bands were isolated and purified from the gel. Subsequently, the purified band was digested with restriction enzymes *Hin*dIII and *Bam*HI. The digested PCR product was then ligated with the p5E-mcs vector, which was digested with the same enzymes using Ligation High (Toyobo). Multisite Gateway cloning [26] was performed using the destination vector pDestTol2pA2, the 5′ entry vector containing the *fabp10* promoter, the middle entry vector containing pME-mCherry, and the 3′ entry vector containing p3E-polyA. DNA constructs (25 pg) and Tol2 mRNA (25 pg) were microinjected into WT zebrafish embryos at the single-cell stage. Six days post-injection, fish were examined using fluorescence microscopy, and mcherry-expressing fish were saved. Germline-integrated transgenic zebrafish were selected from these mcherry-positive fish by raising them to sexual maturity and breeding them with WT zebrafish.

### WES automated simple Western blot assay

Samples were lysed with lysis buffer (0.5% NP-40, 10% glycerin, 50 mM HEPES–KOH [pH 7.8], 150 mM NaCl, and 1 mM EDTA) using protease and a phosphatase inhibitor cocktail (Thermo Fisher Scientific). Protein samples were separated by capillary electrophoresis using 12- 230-kDa Wes Separation Module capillary cartridges in a Simple Protein Wes system (ProteinSimple Wes; ProteinSimple; San Jose, CA, USA), according to the manufacturer's protocol. The following antibodies were used: Lars (#13,868; Cell Signaling Technology, Beverly, MA, USA; 1:50) and β-actin (A3854; Sigma-Aldrich, St. Louis, MO, USA; 1:100). The anti-rabbit and anti-mouse modules for the Wes kit (DM-001 and DM-002; ProteinSimple), which include luminol-S, peroxide, antibody diluent 2, streptavidin-HRP, anti-rabbit secondary antibody, and anti-mouse secondary antibody, were used for detection. The intensities of the acquired chemiluminescence signals were quantified using the AlphaView and Compass software programs (ProteinSimple).

### Morphological analyses

Zebrafish larvae were placed in 3% methylcellulose and images were acquired using a Leica M205 FA fluorescent stereo microscope (Leica, Wetzlar, Germany). Tg[*fabp10*:mcherry] larvae were immobilized in 3% methylcellulose and imaged in vivo using an RFP fluorescence filter. Hepatic structures were traced, and area and circularity were quantified using the ImageJ Fiji software program (1.53t; National Institutes of Health, Bethesda, MD, USA) [27, 28].

### Histopathological staining and fluorescent immunostaining

Histopathological staining and fluorescent immunostaining were performed on paraffin-embedded or frozen sections. For histopathological staining, 5 days post-fertilization (dpf) larvae zebrafish were fixed in 4% paraformaldehyde (PFA) overnight. Tissues were then dehydrated, embedded in paraffin, and sectioned to 5-µm thickness. The samples were initially stained with a hematoxylin solution for 20 s and rinsed with deionized water. They were then stained with eosin solution for 60 s, rinsed again with deionized water, and dehydrated using a series of ascending ethanol concentrations. Excess protein was removed using xylene for 30 s (three repetitions). Finally, the coverslips were mounted using mounting medium. Cryosectioning was performed to obtain samples for immunofluorescence staining. Samples were fixed with 4% PFA for 16 h and then incubated in a microcentrifuge tube with 30% sucrose in phosphate-buffered saline until samples sank down to the bottom of the tube. Samples were then transversally embedded in a mixture of 30% sucrose and Tissue-Tek O.C.T. Compound (4583; Sakura-Finetek, Tokyo) (2:1) and fixed in liquid nitrogen. Sections of 10-µm thickness were obtained using a Leica CM1950 microtome.

An immunofluorescence analysis was performed using the following primary antibodies: anti-p62 (PM045; Medical & Biological Laboratories, Nagoya, Japan) and anti- LC-3 pAb (PM036; Medical & Biological Laboratories). Alexa Fluor 488 donkey anti-rabbit IgG (A21206; Molecular Probes, Eugene, OR, USA; 1:500) was used as the secondary antibody. Images were captured using a laser-scanning microscope (BZ-9000; Keyence, Osaka, Japan).

### Fluorescent staining of accumulated lipids

Fluorescent staining of accumulated lipids was performed on the sections. Zebrafish larvae at 5 dpf were fixed in 4% PFA overnight. Frozen samples were rinsed with phosphate-buffered saline. The samples were then stained with 1 μM Lipi Dye II solution (Funakoshi, Tokyo, Japan) in phosphate-buffered saline and incubated for 1 h at 37 °C. The cells were rinsed three times with phosphate-buffered saline and mounted with fluorescence mounting medium (S3023; Dako, Agilent Technologies). Images were captured using a laser-scanning microscope (BZ-9000; Keyence).

### Bafilomycin A1 and A922500 treatments

In experiments employing autophagy inhibitors, embryos were treated in embryo medium from 72 to 120 h post-fertilization (hpf) for a morphological analysis, with Bufilomycin A1 (2.5 nM; EMD Millipore, Darmstadt, Germany) or dimethyl sulfoxide (DMSO) as a control. For experiments utilizing DGAT1 inhibitors, embryos were similarly treated in embryo medium from 72 to 120 hpf for the morphological analysis, with A922500 (2 mM; Sigma-Aldrich, St. Louis, MO, USA) or DMSO as a control. The medium containing the compounds was changed daily.

### Statistical analyses

Statistical analyses were performed using the GraphPad Prism software version 8 (GraphPad Software, Inc., San Diego, CA, USA). All values are expressed as the mean ± standard error of the mean. Shapiro–Wilk and Brown–Forsythe tests were performed to analyze the normal distribution and homogeneity of the data, respectively. The different groups were compared using the nonparametric independent samples Kruskal–Wallis test for non-normally distributed variables, and the results obtained were expressed as median and interquartile ranges. In contrast, when the data had a normal distribution, they were analyzed using a one-way analysis of variance (ANOVA) followed by Tukey’s pairwise comparison tests. Statistical differences in the survival curves were analyzed using the log-rank (Mantel-Cox) test. Statistical significance was set at *P* < 0.05.

## Results

### An ILFS1 patient with liver dysfunction

**Fig. 1:**
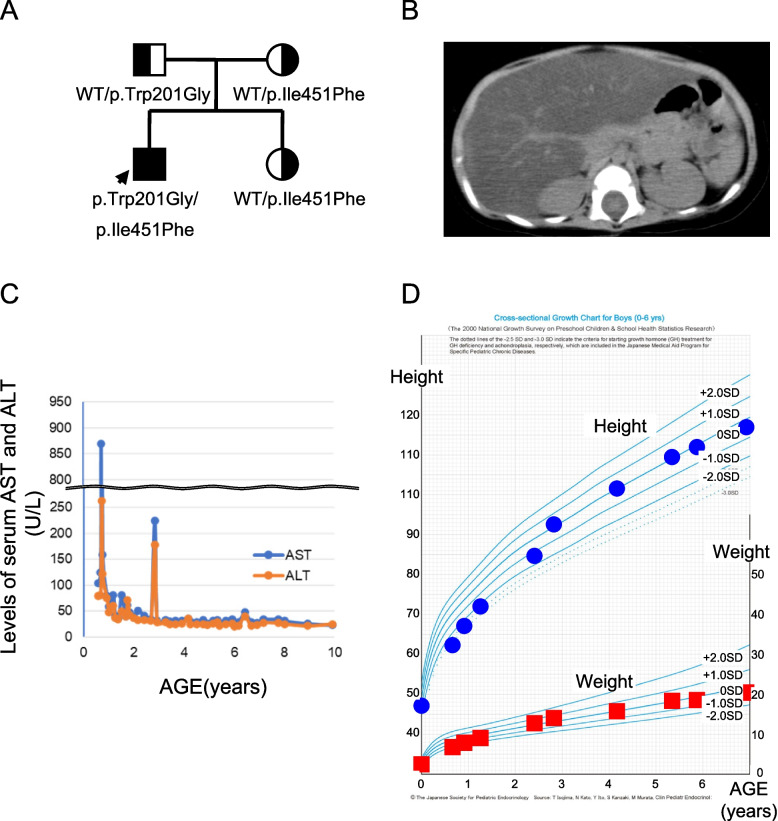
Clinical information of an infantile liver failure syndrome type 1 patient with biallelic *LARS1* variants. **A** Pedigree of the family. **B** Abdominal computed tomography image at eight months old. **C** Changes in serum levels of AST and ALT. **D** Developmental curve

The patient was the first male child born to a non-consanguineous Japanese couple. His younger brother and parents had no congenital abnormalities, including liver disease (Fig. 1A). He was delivered at 37 weeks’ gestation with a birth weight of 2,320 g (9.2%tile). Marked hepatomegaly and failure to thrive were detected during routine checkups by a primary pediatrician at seven months old, and he was referred to our hospital. At 8 months old, his height was 62.2 cm (-3.3 standard deviations [SD]), body weight was 6.6 kg (-2.1 SD), and head circumference was 43.9 cm (0.0 SD). The patient presented with a cherubic face with full cheeks, hepatomegaly (approximately 8 cm below the costa), and mild hypotonia. He was able to control his head by himself but lacked the ability to roll over and sit up unaided. Abdominal computed tomography (CT) revealed a diffuse, low-density, and enlarged liver (Fig. 1B).

Laboratory findings demonstrated mild elevation of serum AST and ALT levels (103 U/l and 70 U/l, respectively) with mild microcytic anemia (hemoglobin 10.6 g/dl, mean corpuscular volume 56.4 fl, mean corpuscular hemoglobin, 17.6 pg). Several days later, he developed a high fever for the first time after birth, which was caused by a human herpesvirus 6 infection. His liver dysfunction soon progressed to ALF as elevation of transaminases (AST 870 U/l, ALT 263 U/l) with reduction of protein synthesis (PT-INR 1.53) and hypoalbumininema (albumin 2.47 g/dl), remarkable anemia (hemoglobin 6.3 g/dl), and thrombocytopenia (platelet count 19,000/μl) (Fig. 1C). He continued to have a fever, generalized edema, oliguria, and respiratory distress and received treatments that included acetaminophen administration, albumin infusion, red blood cell transfusion, and oxygen therapy.

His critical condition recovered with defervescence after five days. Following this episode, he experienced four episodes of febrile illnesses, including acute pharyngitis, hand-foot-mouth disease, and acute gastroenteritis, over the next two years. However, the symptoms appeared to be mild, and ALF did not recur, as transaminase levels peaked at AST 80–220 U/l and ALT 70–260 U/l during these episodes, and growth retardation gradually normalized by 3 years old (Fig. 1C, D). His febrile episodes after his first three years of life included negligible deterioration of the liver function. His psychomotor development progressed normally, with a developmental quotient at 3 years old, as assessed by the Enjohji Developmental Test in Infancy and Early Childhood 106; however, cognitive dysfunction was identified at 6 years old using the Wechsler Intelligence Scale for Children-Fourth edition.

The patient is now 12 years old, and the most recent data are as follows: height, 147.3 cm (-0.2 SD); weight, 34.9 kg (body mass index, 16.1); serum AST level, 24 U/l; serum ALT level, 22 IU/l; serum albumin level 4.04 g/dl; hemoglobin 12.6 g/dl; platelet count, 395,000/μl; and white blood cell count, 6,870/μl, indicating a normal physical growth and liver function with mild anemia.

### LARS1 as a single candidate gene by WES

**Fig. 2:**
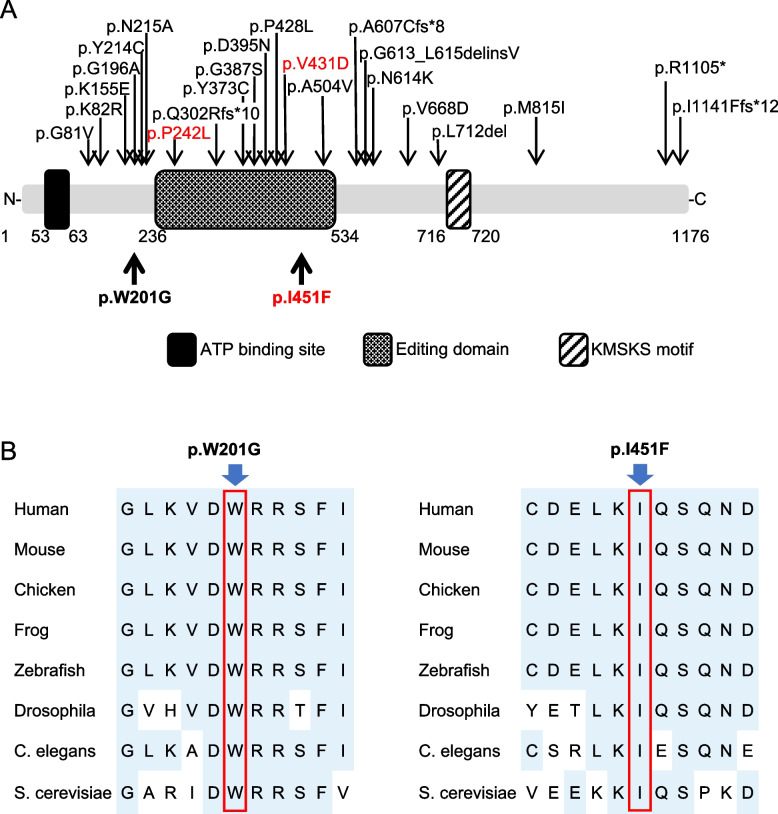
Leucine-tRNA synthetase (LARS) mutations. **A** LARS domains and pathogenic variants found in infantile liver failure syndrome type 1 patients. Variants in our patients are shown in bold. Variants in another reported case with severe manifestation in the neonatal period are in red. **B** Conservations of the missense variant in LARS

WES using the child-parent trio revealed compound heterozygosity in the infant for two potentially pathogenic variants of *LARS1* [NM_020117.9] (Fig. 1A). One missense variant, c.601 T > G; p.Trp201Gly in exon 7 [NM_020117.9], is paternally inherited and has not been previously reported in ClinVar. An in silico analysis suggested that p.Trp201Gly probably damaged the protein structure and/or function (Polyphen2: score 1.000; probably damaging (Supplementary Table S1). Another missense variant, c.1351A > T; p.Ile451Phe in exon 14 [NM_020117.9], is maternally inherited and has been previously described in a Japanese patient with ILFS1 [29]. It was located in the LARS-editing domain (Fig. 2A). Importantly, 9 of the 23 pathogenic variants previously reported in ILFS1 patients were located in this domain. Three editing-domain variants, including p.Ile451Phe, show severe symptoms during the neonatal period [1, 3, 4, 29–31]. An in silico analysis predicted that p.Ile451Phe was also probably damaging to the protein structure and/or function (PolyPhen-2:0.921; probably damaging) (Supplementary Table S1). Notably, both missense variants affected the evolutionarily conserved residues (Fig. 2B).

### Liver defects in larsb-I451F zebrafish during liver development

**Fig. 3:**
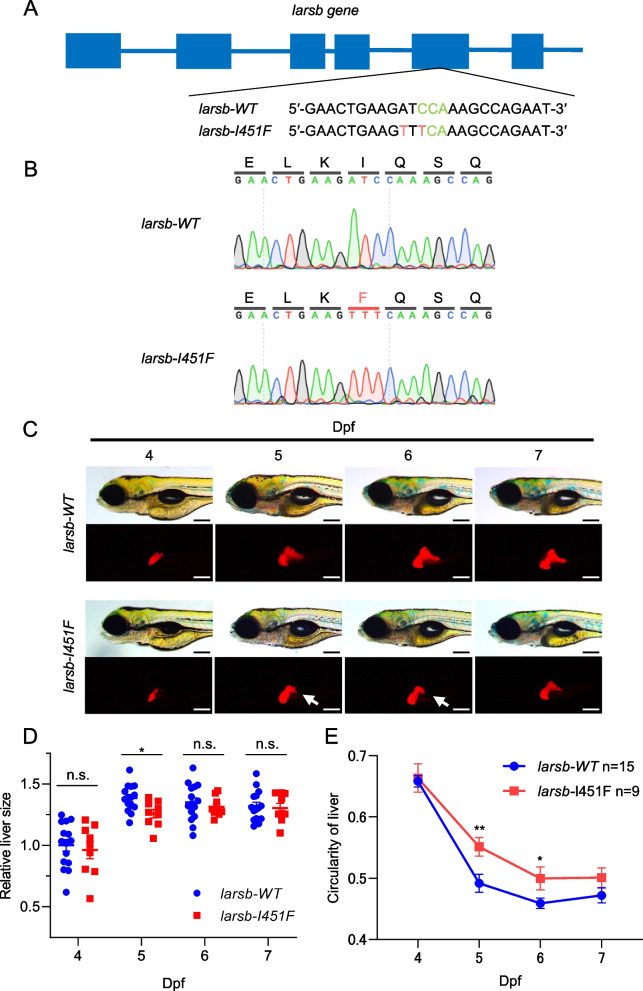
*Larsb*-knockin larvae display liver abnormality during the liver developmental stage. **A** Diagram showing the *larsb* genomic locus and *larsb*-knockin (*larsb-I451F*) zebrafish mutant genotype. **B** In the genomic sequencing analysis chromatograms, the mutation site in the *larsb-I451F* zebrafish is shown in red. **C** Morphological abnormality at 4 to 7 dpf in the livers of *larsb-I451F* larvae with a Tg[*fabp10*:mcherry] background. White arrows indicate the loss of liver edges in *larsb-I451F* larvae. Scale bar: 200 μm. **D** Relative liver area in *larsb-WT* (*n* = 15 fish) and *larsb-I451F* (*n* = 9 fish) larvae with a Tg[*fabp10*:mcherry] background (4 to 7 dpf). *P < 0.05. **E** Circularity of liver in *larsb-WT* (*n* = 15 fish) and *larsb-I451F* (*n* = 9 fish) larvae with a Tg[*fabp10*:mcherry] background (4 to 7 dpf). Error bars indicate SEM. *P < 0.05, **P < 0.01

To assess the pathological relevance of *LARS1* variants in the liver, we generated A-to-T at codon 1351 and C-to-T at codon 1353 knock-in zebrafish lines using CRISPR/Cas9. To obtain more efficient knock-in using genome editing, we replaced the two bases that changed the PAM sequence (Fig. 3A). Among the pathogenic variants in the *LARS1* gene (p.Trp201Gly/p.Ile451Phe) identified in our patient, we focused on the p.Ile451Phe variant, which has been found in other Japanese patients, suggesting a Japanese founder effect and is located within the editing domain of the LARS protein, where pathological variants have accumulated [29]. We designed a model of the *larsb* p.Ile451Phe mutation (*larsb-I451F*) to elucidate the pathogenesis of ILFS1 (Fig. 3B).

First, we measured the amount of Lars protein in the whole body of *larsb-I451F* zebrafish larvae. Western blotting confirmed that the amount of Lars protein in *larsb-I451F* zebrafish was similar to that in WT *larsb* zebrafish (Supplementary Figure S1A-B). Patients with ILFS1 exhibit hepatomegaly and liver damage with rapid progression after viral infection during neonates and infancy [3]. To analyze the morphology of the liver, *larsb-I451F* zebrafish were crossed with Tg[*fabp10*:mcherry] transgenic zebrafish, which constitutively express mCherry fluorescent protein in the liver [21, 22]. Because zebrafish livers mature at the larval stage by five days old [32, 33], we observed *larsb-I451F* zebrafish livers at approximately 5 dpf. At 5 dpf, *larsb-I451F* zebrafish exhibited a significant decrease in the liver area and an increase in liver circularity (Fig. 3C-E), which are common features of liver diseases [27]. As *larsb-I451F* zebrafish grew, abnormalities in liver area and circularity gradually improved by 7 dpf (Fig. 3C-E). In addition, *larsb-I451F* zebrafish survived to adulthood in the same manner as WT zebrafish. Thus, we found morphological abnormalities that predominantly appeared in the developing hepatocytes of *larsb-I451F* zebrafish.

### Hepatic adiposity in larsb-I451F zebrafish

**Fig. 4:**
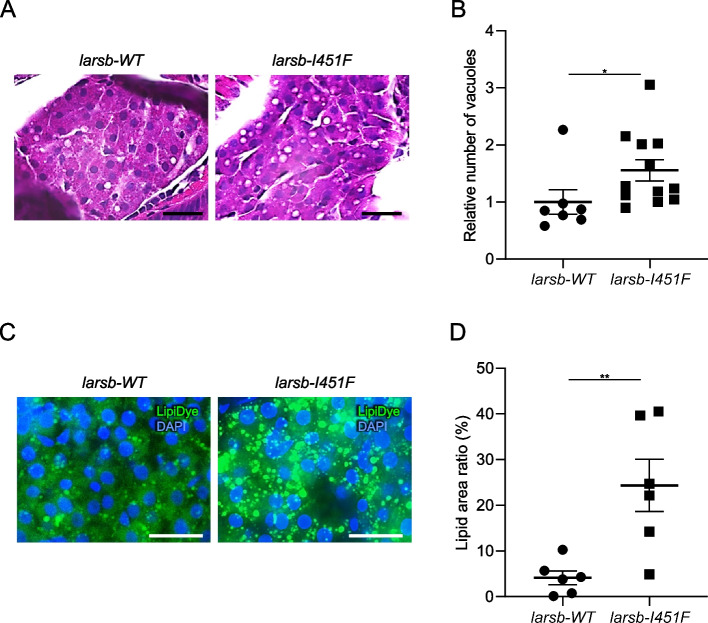
Histopathology and lipids staining of the liver in *larsb*-knockin larvae. **A** Hematoxylin and eosin staining of the liver in *larsb*-knockin (*larsb-I451F*) larvae at 5 dpf. Scale bar: 20 μm. **B** Quantification of the vesicle number in *larsb-WT* (*n* = 7 sections) and *larsb-I451F* (*n* = 12 sections) liver at 5 dpf. Error bars indicate SEM. *P < 0.05. **C** Lipids staining of the liver in *larsb-I451F* larvae at 5 dpf. Scale bar: 20 μm. **D** Quantification of the lipid area in *larsb-WT* (*n* = 6 sections) and *larsb-I451F* (*n* = 6 sections) larvae liver at 5 dpf. Error bars indicate SEM. **P < 0.01. Dpf: days post fertilization

The liver was histopathologically analyzed. The livers of *larsb-I451F* larvae contained more vacuoles than those of *larsb-WT* larvae (Fig. 4A, B). Multiple vacuoles in the cytoplasm and clear circular spaces with sharp outlines and contours are characteristic of fat-type vacuolation [34].

To examine whether or not intrahepatic vacuoles in *larsb-I451F* zebrafish were lipid droplets, we evaluated intrahepatic lipids using fluorescent staining [35]. Many lipid droplets visualized by lipid dye droplet staining were observed in the livers of *larsb-I451F* larvae compared to those of *larsb-WT* larvae (Fig. 4C, D). These data indicate that LARS dysfunction induces hepatic lipid droplet formation. Most patients with ILFS1 present liver steatosis [3]. Thus, *larsb-I451F* zebrafish exhibited a phenotype analogous to that observed for ILFS1, indicating that the function of LARS in the liver is conserved between zebrafish and humans.

### The larsb-I451F mutation augments autophagy in liver

**Fig. 5:**
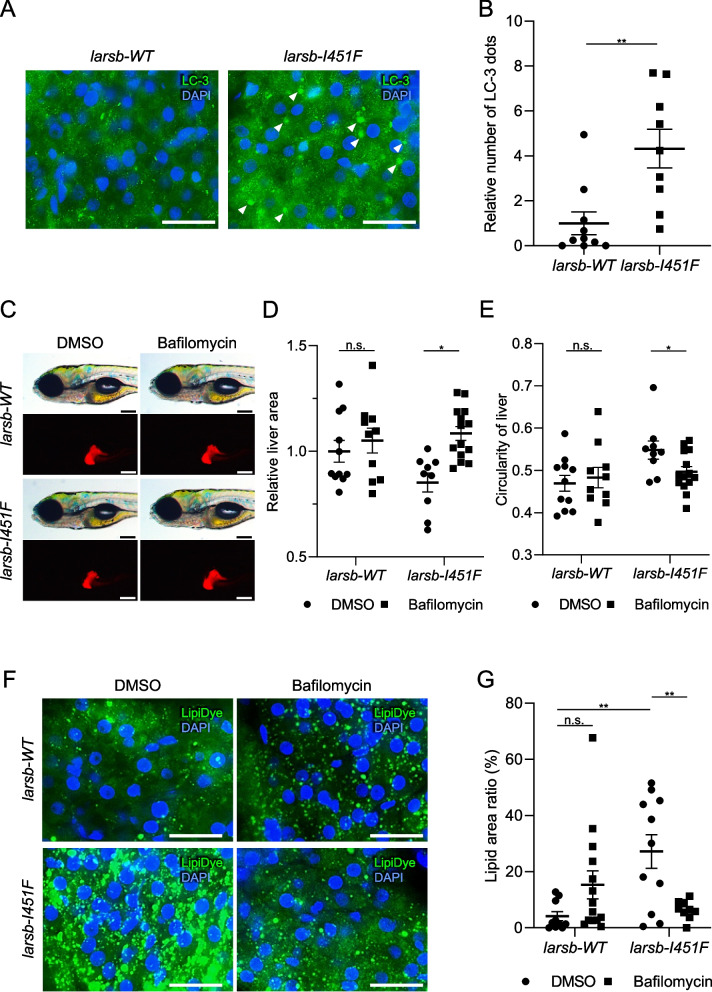
Enhanced autophagy in the liver of *larsb*-knockin larvae. **A** Immunostaining of LC-3 in the liver of *larsb*-knockin (*larsb-I451F*) larvae at 5 dpf. Scale bar: 20 μm. White arrowheads indicate LC3-positive dots. **B** Quantification of the number of LC-3 dots in *larsb-WT* (*n* = 10 sections) and *larsb-I451F* (*n* = 9 sections) larvae liver at 5 dpf. Error bars indicate SEM. **P < 0.01. **C** Morphological abnormality in the livers of *larsb-I451F* larvae at 5 dpf with a Tg[*fabp10*:mcherry] background treated with DMSO or bafilomycin. Scale bar: 200 μm. **D** Liver area in *larsb-I451F* larvae at 5 dpf with a Tg[*fabp10*:mcherry] background treated with DMSO (*larsb-WT n* = 11 sections, *larsb-I451F n* = 9 sections) or bafilomycin (*larsb-WT n* = 10 sections, *larsb-I451F n* = 14 sections). Error bars indicate SEM. *P < 0.05. **E** Circularity of the liver in *larsb-I451F* larvae at 5 dpf with a Tg[*fabp10*:mcherry] background treated with DMSO (*larsb-WT n* = 11 sections, *larsb-I451F n* = 9 sections) or bafilomycin (*larsb-WT n* = 10 sections, *larsb-I451F n* = 14 sections). Error bars indicate SEM. *P < 0.05. **F** Lipids staining of the liver in *larsb-I451F* larvae at 5 dpf treated with DMSO or bafilomycin. Scale bar: 20 μm. **G** Quantification of the lipid area in *larsb-I451F* larvae liver at 5 dpf treated with DMSO (*larsb-WT n* = 10 sections, *larsb-I451F n* = 11 sections) or bafilomycin (*larsb-WT n* = 14 sections, *larsb-I451F n* = 9 sections). Error bars indicate SEM. **P < 0.01. Dpf: days post fertilization

Excessive activation of autophagy has been observed in *larsb*-deficient zebrafish [19]. Therefore, to assess whether or not autophagy is involved in liver abnormalities in *larsb-I451F* zebrafish, we evaluated the status of autophagy using fluorescent immunostaining for LC-3 and p62 in *larsb-I451F* larvae. LC-3, a downstream component of the autophagy pathway that participates in autophagosome formation, is widely used to monitor autophagy [36]. While the expression of p62, a selective autophagy substrate, did not differ markedly between *larsb-I451F* and *larsb-WT* larvae (Supplementary Figure S2A-B), many autophagosomal structures visualized with LC-3 were observed in the livers of *larsb-I451F* larvae compared to *larsb-WT* larvae (Fig. 5A, B). Therefore, LARS dysfunction appears to enhance autophagy in developing livers.

Next, to validate whether or not the lipid droplets detected in the livers of *larsb-I451F* larvae were induced by enhanced autophagy, *larsb-I451F* larvae were treated with an inhibitor specific to diacylglycerol acyltransferase 1 (DGAT1) (A922500). DGAT1 and DGAT2 mediate the final step in the synthesis of triacylglycerols from fatty acids stored in lipid droplets [37, 38]. Because both DGAT1 and DGAT2 act on liver lipid droplet formation due to overnutrition, inhibition of DGAT1 alone does not usually improve lipid droplets [37–39]. In contrast, hepatic lipid accumulation via autophagy is specifically mediated by DGAT1 [39]. We demonstrated that A922500 treatment improved the accumulation of intrahepatic lipids in *larsb-I451F* larvae (Supplementary Figure S3A-B). Consequently, it was likely that the accumulated lipid droplets in the livers of *larsb-I451F* zebrafish had been induced by autophagy.

To verify whether or not liver abnormalities in *larsb-I451F* larvae were due to excessive autophagy, we treated *larsb-I451F* larvae with the autophagy inhibitor, bafilomycin A1. Bafilomycin treatment improved the abnormal liver area and decreased liver circularity in *larsb-I451F* larvae at 5 dpf (Fig. 5C-E). The accumulation of intrahepatic lipids was significantly reduced by bafilomycin treatment (Fig. 5F-G). We concluded that hyperactivated autophagy induced by *larsb-I451F* was responsible for liver steatosis.

## Discussion

In this study, we report a patient with deleterious variants in *LARS1* gene and demonstrate the pathogenesis of ALF in ILFS1 by excessive autophagy during LARS dysfunction. Our patient presented with intrauterine growth retardation, failure to thrive, developmental delay, microcytic anemia, decreased muscle tone, and acute liver failure in infancy, exaggerated by infections. CT during infancy suggested the accumulation of hepatic fat. Liver dysfunction leading to liver failure, along with growth impairment, psychomotor developmental delay, anemia, and muscular hypotonia, mostly improved by three years old.

Liver dysfunction was most prominent in ILFS1 patients during infancy, which aligns with the finding of this study that *larsb-I451F* zebrafish exhibited liver abnormalities during the developmental stage. A histopathological analysis of *larsb-I451F* zebrafish showed the accumulation of lipid droplets in the liver, which mimicked the liver of ILFS1 patients caused by biallelic variants of the human *LARS1* gene. In addition, enhanced autophagy was observed in the livers of *larsb-I451F* zebrafish. Inhibition of DGAT1, which converts fatty acids to triacylglycerols, improves lipid droplets in the liver of *larsb-I451F* zebrafish. Furthermore, the inactivation of autophagy by bafilomycin treatment significantly decreased the accumulation of intrahepatic lipids. These results suggest that LARS dysfunction in ILFS1 induces steatosis in the developing zebrafish liver via enhanced autophagy, pointing to the potential treatment of ALF by inhibiting autophagy.

ILFS1 patients demonstrate liver failure during infancy and episodes of febrile illness [1]. Hepatic dysfunction in these patients is characterized by recurrent liver crises with elevated liver enzyme levels as a prominent clinical manifestation during the acute phase [3]. Liver crises are most severe in infancy and tend to improve with age [1]. Histopathological findings in the liver are characterized by hepatocellular damage resembling acute liver failure, steatosis, fibrosis, and cirrhosis, consistent with the clinical features of recurrent acute and chronic liver failure [3]. The hepatic dysfunction in our ILFS1 patients was quite similar to that in previous reports, indicating a large impact of LARS dysfunction on infantile liver tissue.

In our previous study, *larsb*-knockout zebrafish exhibited progressive liver failure, anemia, and neurological defects that resembled the symptoms of patients with ILFS1 [19]. However, the livers of *larsb*-knockout zebrafish exhibited cytoplasmic loss due to severe damage, and early lethality precluded a further histological examination [19]. In the present study, we demonstrated the accumulation of lipids through enhanced autophagy in the liver of *larsb-I451F* zebrafish larvae. Although *larsb-I451F* zebrafish had the same amount of LARS protein as *larsb-WT* zebrafish, pathological variants of *LARS1* led to a reduction in the aminoacylation activity of LARS, as previously reported in fibroblasts from ILFS1 patients [3]. Aminoacylation is executed through the precise functions of leucine sensing and binding to the LARS protein, ATP binding, and structural alterations in LARS [40, 41].

Pathogenic variants of *LARS1* that exhibit abnormalities in any of these functions lose their capacity to stimulate the mTORC1 pathway, which regulates autophagy [42]. Autophagy serves as an alternative energy source during nutrient deficiency by facilitating the breakdown of cellular components to produce fatty acids [43, 44]. However, excessive enhancement of autophagy beyond physiological limits can lead to autophagic cell death [45], which has also been confirmed in *larsb*-knockout zebrafish. While moderate autophagy serves as a protective mechanism against cell death during starvation, the surplus fatty acids generated during this process can be toxic and need to be directed into the mitochondria and used for energy production or stored as lipid droplets through DGAT1-mediated pathways [39, 46], as shown by *larsb-I451F* zebrafish in this study. Recent studies using mouse models have reported that increased autophagy promotes lipid release from adipocytes throughout the body. This process involves accelerated degradation of stored fats within adipocytes, resulting in the release of free fatty acids into the bloodstream. These fatty acids are subsequently transported to the liver, where they accumulate as fat deposits, exacerbating hepatic steatosis [47, 48]. It is therefore plausible that upregulated autophagy due to LARS dysfunction in *larsb-I451F* zebrafish induces fatty acid release from adipocytes, potentially contributing to hepatic lipid accumulation mediated by DGAT1. The dysregulation of liver autophagy might differ between cases with complete deficiency and those with partially retained function of LARS. Severe phenotypes in *larsb*-knockout zebrafish emerge because of the absence of functionalities, such as leucine binding, aminoacylation, and the C-terminal region interacting with mTORC1 [7]. In *larsb-I451F* zebrafish harboring the pathogenic variant p.Ile451Phe, which encodes an editing domain responsible for removing erroneously incorporated amino acids, the precise charging of leucine is impeded [49]. Consequently, accurate sensing of the intracellular leucine concentration is compromised, likely leading to the promotion of autophagy as a response to perceived amino acid deficiency. Further analyses using knock-in zebrafish with other genotypes will help elucidate the mechanism by which LARS dysfunction activates autophagy.

*Larsb-I451F* zebrafish exhibited an atypical liver morphology at 5 dpf. In zebrafish embryogenesis, critical organ systems, such as the liver, rapidly develop at 5 dpf [50]. During this process, the complex mechanism of autophagy plays a crucial role in regulating cellular proliferation and differentiation. In zebrafish embryo development, autophagic activity sufficiently increased from 3 to 5 dpf [51, 52]. In patients diagnosed with ILFS1, ALF is predominantly observed in the neonatal and infantile phases [3]. While ILFS1 patients commonly present with hepatomegaly, the liver size in *larsb-I451F* zebrafish was diminished at the larval stage. It is estimated that the liver size can show either enlargement or reduction depending on the degree of damage and stage of liver disease [53]. The need for efficient resource recycling during the zebrafish larval stage may have also resulted in more accelerated autophagy than that in humans, resulting in a smaller liver size. Given the resemblance between clinical liver pathology in ILFS1 patients and histopathological findings in *larsb-I451F* zebrafish larvae, it is plausible that liver damage is predominantly observed in neonates and infants owing to defects in the *LARS1* gene caused by increased autophagy. We further postulate that if remarkable and specific stimuli activate autophagy in cells, organ-specific damage can occur at any time during their lifespan.

Our findings suggest that dysregulation of autophagy, caused by biallelic pathogenic variants of *LARS1* leads to liver steatosis. Since significant similarities were observed between the liver tissues of human ILFS1 and those of *larsb-I451F* zebrafish, this knock-in zebrafish more closely replicates ILFS1 than does the *larsb*-knockout zebrafish. While patients with ILFS1 have a reduced risk of ALF after infancy, neurological and hematopoietic complications may relapse or appear in the long term. Unlike *larsb*-knockout larvae, *larsb-I451F* larvae can survive for as long as adult zebrafish, so a straightforward evaluation of neurodevelopment and hematopoiesis can be achieved. Inborn errors of metabolism, such as Niemann-Pick disease type C and Gaucher disease, are known to present with distinct hepatic abnormalities during infancy and neurological symptoms in adolescence or adulthood. Similarly, citrin deficiency, which causes transient cholestatic liver disease in infancy, suddenly manifests as hyperammonemia in later adulthood, after a long asymptomatic period. Consequently, long-term clinical trajectories can only be elucidated using model organisms that are capable of tolerating long-term observation. Previous case reports of ILFS1 are limited in number, and the long-term clinical characteristics of surviving cases remain unclear. Zebrafish offer advantages as a suitable model organism for such observations and screening for potential drugs or chemical compounds. *Larsb-I451F* zebrafish may serve as an optimal model for long-term study of ILFS1 and may provide invaluable findings for further basic and clinical research.

## Conclusion

Our study verified that biallelic *LARS1* variants cause liver abnormalities at the developmental stage through an examination of a Japanese ILFS1 patient and *larsb-I451F* zebrafish. A histological examination of the liver in *larsb-I451F* zebrafish revealed that accelerated hepatic autophagy can cause hepatic steatosis. In addition, *larsb-I451F* zebrafish exhibited a long survival in contrast to *larsb*-knockout zebrafish, suggesting its potential utility in dissecting the pathology of ILFS1 impairments in adulthood.

### Supplementary Information

Additional file 1: Table S1. Segregated variants in the family.

Additional file 2: Figure S1. WES automated a simple Western blot assay of Larsb protein expression in *larsb*-knockin zebrafish. (A) Larsb protein capillary-based Western blot images of wild-type and *larsb*-knockin (*larsb-I451F*) zebrafish at 5 dpf. Lanes 1: molecular marker, Lanes 2–3: assay with anti-Larsb antibody, Lanes 4–5: assay with anti-β-actin antibody. β-actin served as the loading control. (B) Densitometric quantification of the relative ratio of Larsb to β-actin in three independent experiments. Number (n) = 3 zebrafish/group. Error bars indicate SEM. *P < 0.05. Dpf: days post fertilization.

Additional file 3: Figure S2. The evaluation of p62 in the liver of *larsb*-knockin larvae. (A) Immunostaining for p62 in the livers of wild-type (*larsb-WT*) and *larsb*-knockin (*larsb-I451F*) larvae at 5 dpf. Scale bar: 20 μm. (B) Quantification of the number of p62 dots in the livers of *larsb-WT* (*n* = 5 sections) and *larsb-I451F* (*n* = 8 sections) larvae at 5 dpf. Error bars indicate SEM. Dpf: days post fertilization.

Additional file 4: Figure S3. Inhibition of DGAT1 prevents the liver steatosis in *larsb*-knockin larvae. (A) Lipid staining of the liver in wild-type (*larsb-WT*) and *larsb*-knockin (*larsb-I451F*) larvae at 5 dpf treated with DMSO or A922500. Scale bar: 20 μm. (B) Quantification of the lipid area in the livers of *larsb-WT* and *larsb-I451F* larvae at 5 dpf treated with DMSO (*larsb-WT n* = 9 sections, *larsb-I451F n* = 8 sections) or A922500 (*larsb-WT n* = 7 sections, *larsb-I451F n* = 8 sections). Error bars indicate SEM. *P < 0.05. DGAT1, diacylglycerol acyltransferase 1; dpf, days post fertilization.

## Data Availability

Original data and materials were obtained upon reasonable request from the corresponding author.

## Abbreviations

ALF: Acute liver failure
WES: Whole-exome sequencing
LARS: Leucyl-tRNA synthetase
ILFS1: Infantile liver failure syndrome type 1
mTORC1: Mammalian target of rapamycin complex 1
WT: Wild-type
Dpf: Days post-fertilization
DGAT1: Diacylglycerol acyltransferase 1
