# Supplementary figures and images for: Biallelic variants in LARS1 induce steatosis in developing zebrafish liver via enhanced autophagy
Source: Orphanet J Rare Dis. 2024 May 28;19:219. doi: 10.1186/s13023-024-03226-6 (PMC11134648; doi:10.1186/s13023-024-03226-6)

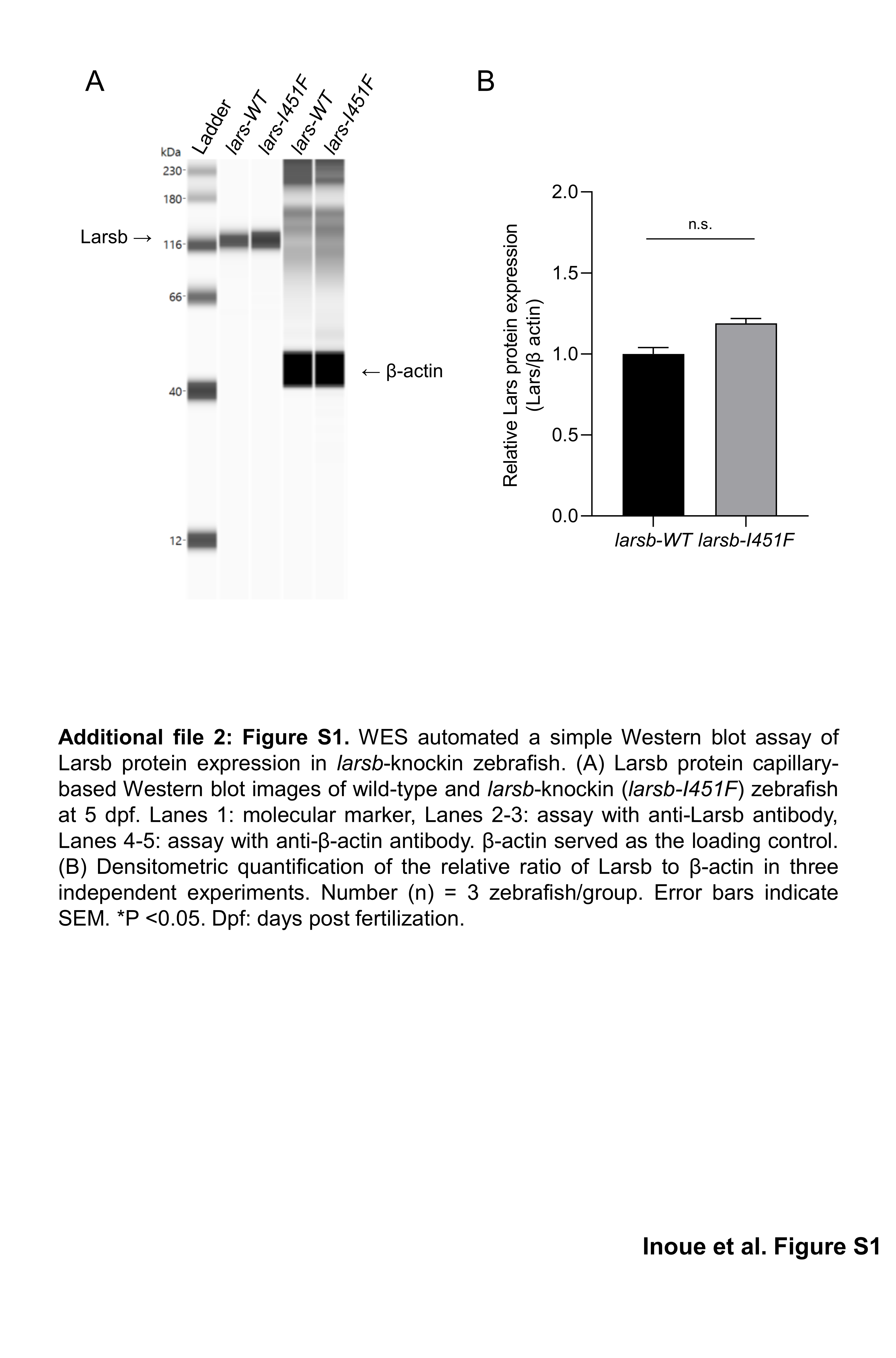

Supplement: Supplementary file 2 — Additional file 2: Figure S1. WES automated a simple Western blot assay of Larsb protein expression in larsb-knockin zebrafish. (A) Larsb protein capillary-based Western blot images of wild-type and larsb-knockin (larsb-I451F) zebrafish at 5 dpf. Lanes 1: molecular marker, Lanes 2–3: assay with anti-Larsb antibody, Lanes 4–5: assay with anti-β-actin antibody. β-actin served as the loading control. (B) Densitometric quantification of the relative ratio of Larsb to β-actin in three independent experiments. Number (n) = 3 zebrafish/group. Error bars indicate SEM. *P < 0.05. Dpf: days post fertilization. [file 13023_2024_3226_MOESM2_ESM.tif]

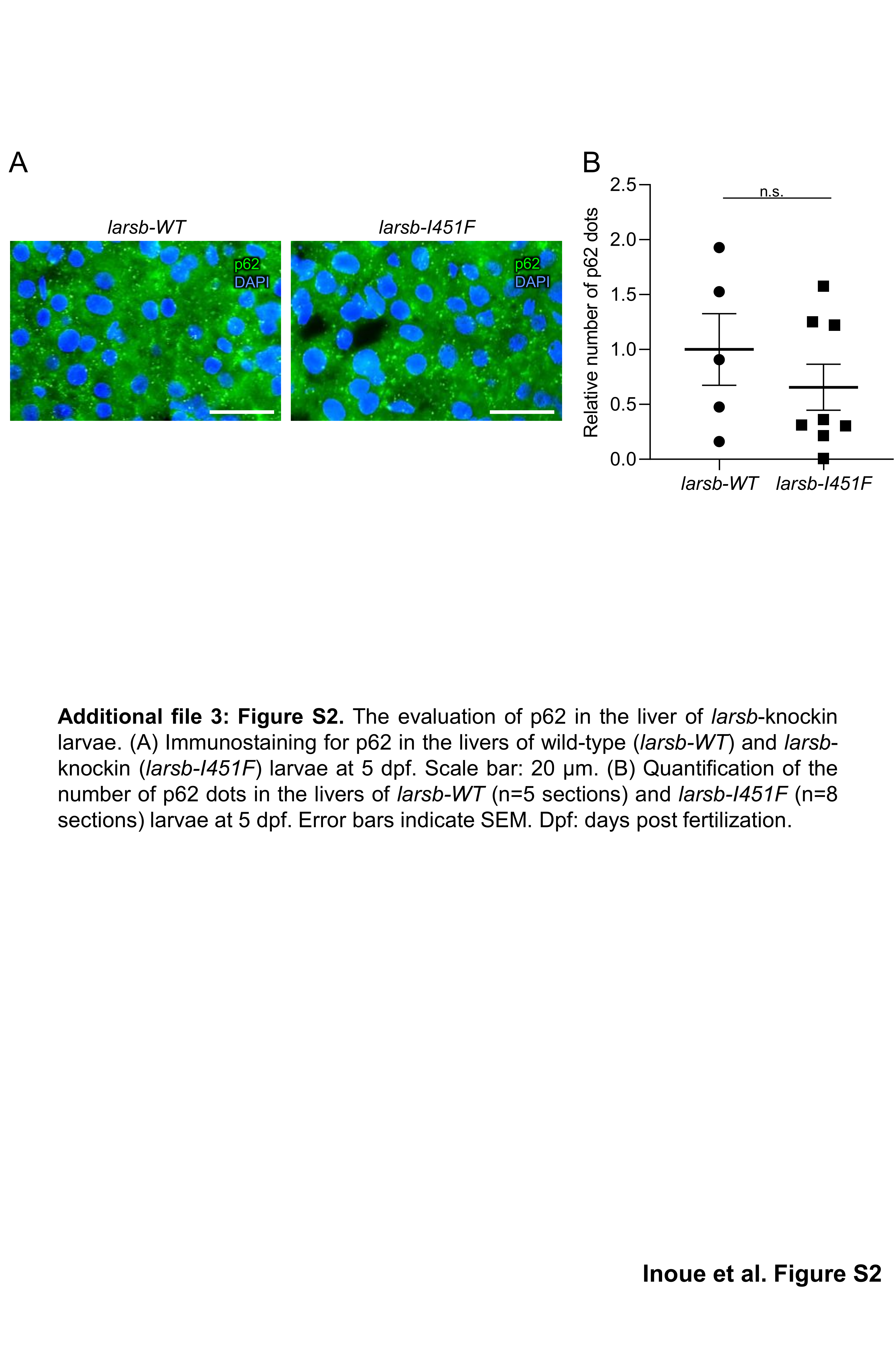

Supplement: Supplementary file 3 — Additional file 3: Figure S2. The evaluation of p62 in the liver of larsb-knockin larvae. (A) Immunostaining for p62 in the livers of wild-type (larsb-WT) and larsb-knockin (larsb-I451F) larvae at 5 dpf. Scale bar: 20 μm. (B) Quantification of the number of p62 dots in the livers of larsb-WT (n = 5 sections) and larsb-I451F (n = 8 sections) larvae at 5 dpf. Error bars indicate SEM. Dpf: days post fertilization. [file 13023_2024_3226_MOESM3_ESM.tif]

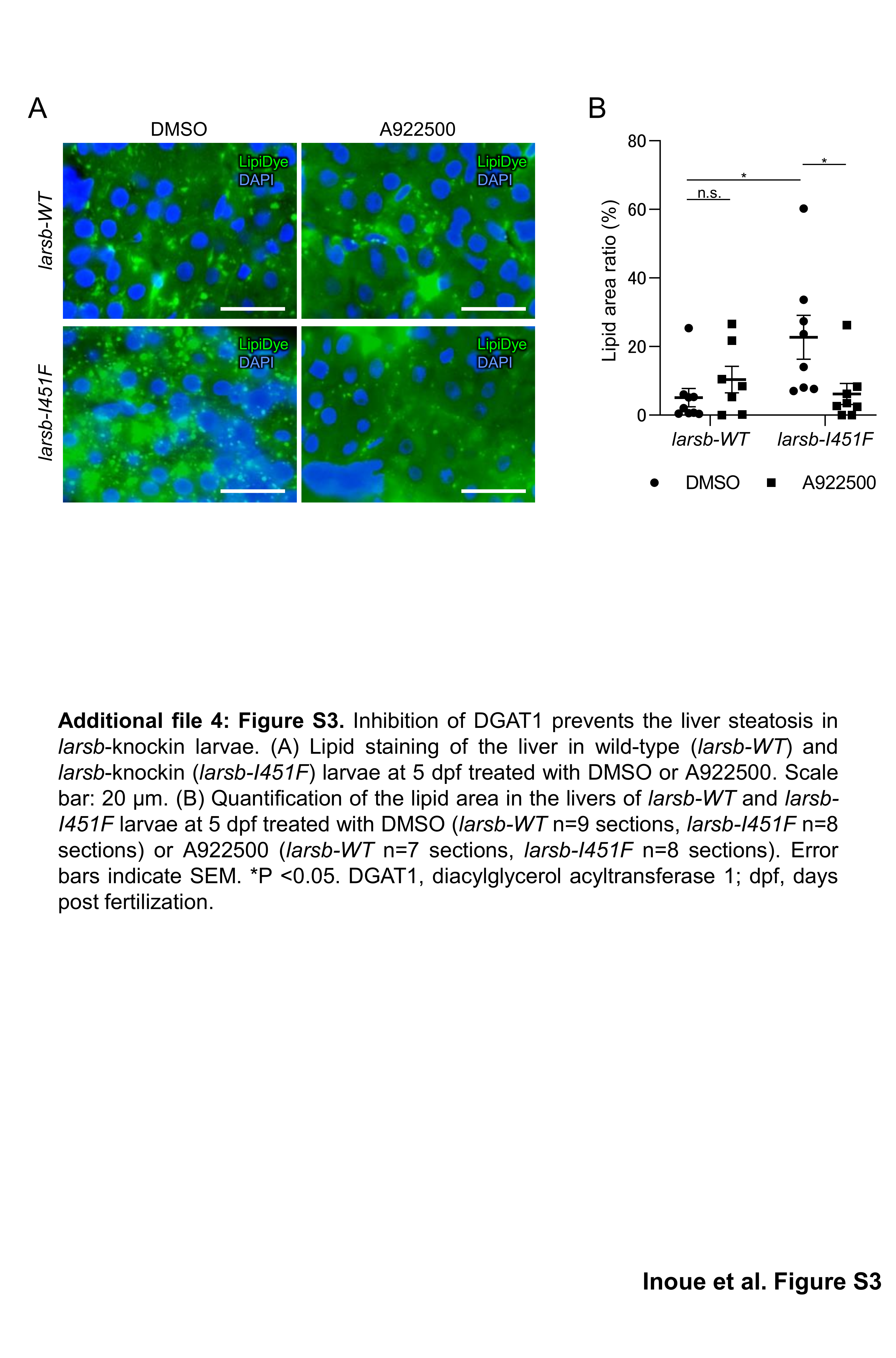

Supplement: Supplementary file 4 — Additional file 4: Figure S3. Inhibition of DGAT1 prevents the liver steatosis in larsb-knockin larvae. (A) Lipid staining of the liver in wild-type (larsb-WT) and larsb-knockin (larsb-I451F) larvae at 5 dpf treated with DMSO or A922500. Scale bar: 20 μm. (B) Quantification of the lipid area in the livers of larsb-WT and larsb-I451F larvae at 5 dpf treated with DMSO (larsb-WT n = 9 sections, larsb-I451F n = 8 sections) or A922500 (larsb-WT n = 7 sections, larsb-I451F n = 8 sections). Error bars indicate SEM. *P < 0.05. DGAT1, diacylglycerol acyltransferase 1; dpf, days post fertilization. [file 13023_2024_3226_MOESM4_ESM.tif]
